# Severe *Babesia microti* Infection in an Immunocompetent Host in Pennsylvania

**DOI:** 10.1177/2324709616663774

**Published:** 2016-08-22

**Authors:** Jeffrey Genda, Elizabeth A. Negron, Mona Lotfipour, Samyuktha Balabhadra, Diana S. Desai, David W. Craft, Michael Katzman

**Affiliations:** 1 PinnacleHealth Hospital System, Harrisburg, PA, USA; 2 Pennsylvania Department of Health, Harrisburg, PA, USA; 3 Penn State College of Medicine, Hershey, PA, USA; 4 Penn State Milton S. Hershey Medical Center, Hershey, PA, USA

**Keywords:** *Babesia microti*, babesiosis, *Ixodes scapularis*, *Borrelia burgdorferi*, Lyme disease

## Abstract

Babesiosis, due to infection by a tick-borne protozoan (predominantly *Babesia microti* in North America), is an emerging health risk that is expanding into new areas and may be unfamiliar to clinicians in locations not previously considered endemic. Manifestations of infection can range from asymptomatic to life threatening, with severe disease more likely in those who have had a splenectomy, are immunocompromised, have chronic medical conditions, or are over 50 years of age. In this article, we describe an elderly but otherwise healthy man from an area not generally considered endemic for babesiosis who presented with severe hemolysis, acute renal failure, and high-level *Babesia microti* parasitemia; serological results suggestive of possible coinfection by *Borrelia burgdorferi* (the agent of Lyme disease, which is carried by the same tick as is *Babesia microti*) also was found. This report highlights that severe babesiosis can occur in an apparently normal host and underscores the continued geographic expansion of this pathogen and the need for early recognition and therapy.

## Introduction

Babesiosis, a malaria-like protozoan infection caused predominantly by *Babesia microti* in North America, is an emerging health risk.^1^ *B microti* is transmitted by the *Ixodes scapularis* tick, with the highest incidence of human infection in the Northeastern and upper Midwest states.^2^ Most infections occur in individuals from endemic areas, and in healthy persons the clinical course is often mild and self-limited. However, severe disease with organ damage and death can occur in certain individuals, particularly those who have had a splenectomy, are immunocompromised, have major medical comorbidities, or are over the age of 50 years.^1,3^ Our otherwise healthy but older patient, who was not from an area historically considered endemic for babesiosis, presented with hemolysis, acute renal failure, and a high parasitemia level. Concurrent infection with *B burgdorferi* also was suggested by serological studies. This case supports other reports of severe babesiosis in immunocompetent hosts^4-6^ and underscores the continued geographic expansion of babesiosis.

## Case Description

**Figure 1.**
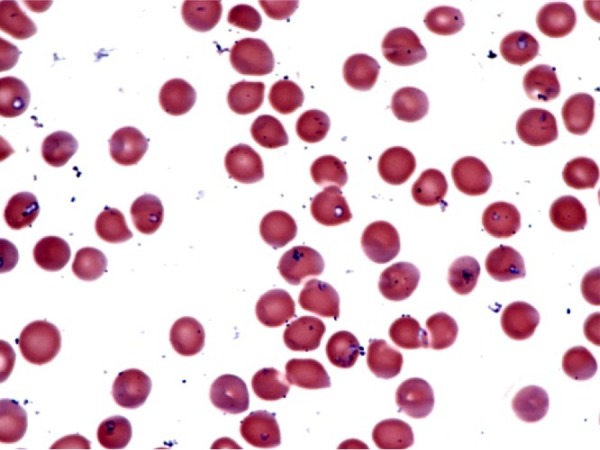
Peripheral smear from hospital day 1 showing intracellular ring forms consistent with *Babesia microti* in approximately 10% to 20% of red blood cells; extracellular parasites also are evident. Wright-Giemsa stain, original magnification 1000×.

A 75-year-old man presented to the hospital in early August 2015 after noticing that his urine had become dark for several days. He had a history of hypertension, hyperlipidemia, and tobacco use but was active on his farm in Berks County in southeastern Pennsylvania. Near the end of July he had washed several ticks off his legs but did not think any had been embedded. Several days later he noticed dark urine, which persisted, and he sought care at a hospital in southcentral Pennsylvania, just west of his county of residence. On initial assessment, he was afebrile with normal vital signs, and his exam was remarkable for scleral icterus and a palpable spleen tip. Laboratory evaluation revealed a leukocyte count of 5.8 × 10^9^ cells/L (normal range 4.5-10.0 × 10^9^ cells/L), anemia with a hemoglobin of 10.4 g/dL (normal 13-16 g/dL), thrombocytopenia with a platelet count of 41 × 10^9^/L (normal 150-350 × 10^9^/L), acute kidney injury with creatinine 3.1 mg/dL compared with his baseline creatinine of 1.4 mg/dL (normal 0.5-1.6 mg/dL), elevated bilirubin at 10.6 mg/dL (normal 0.2-1.5 mg/dL), and reduced haptoglobin of 4 mg/dL (normal 36-195 mg/dL) consistent with hemolysis. The initial peripheral blood smear was reported as showing intraerythrocytic ring forms consistent with *Babesia* (Figure 1). Antimicrobial therapy directed against *Babesia* was initiated with atovaquone plus azithromycin, and the patient was transferred to an academic referral hospital for more specialized care.

On arrival at the referral hospital, his laboratory values worsened, with a hemoglobin of 7.6 g/dL, creatinine 4.7 mg/dL, and bilirubin 19.7 mg/dL; an elevated reticulocyte count of 13.5% was documented (normal range 0.4% to 2.1%). A peripheral smear was reported as showing approximately 40% to 50% parasitemia, as well as many extracellular parasites. An apheresis catheter was placed and the patient underwent a red cell exchange of 2 blood volumes, with subsequent serial parasite levels of 2%, 1%, <1%, and negative. His antimicrobials were changed to clindamycin plus quinine (with administration of one dose of quinidine until quinine became available). Given concern for concomitant tick-borne infections, empiric doxycycline was added. Abdominal imaging confirmed an intact but mildly enlarged spleen. At the time of discharge (hospital day 15), he remained anemic but his platelet count was normal and his creatinine and bilirubin had fallen to 1.5 and 2.3 mg/dL, respectively. He finished approximately 10 to 14 days of each antimicrobial and was well at 2 weeks and at 4 months after discharge. A follow-up blood smear 6 weeks after hospital discharge showed no parasites.

## Microbiological Testing

Laboratory test results (some of which became available after hospital discharge) confirmed acute infection by *B microti* both serologically, with positive tests for IgM antibodies and IgG antibodies (each at a titer of 1:320 by immunofluorescence; Laboratory Corporation of America, Dublin, OH), as well as by a positive real-time polymerase chain reaction (PCR) assay from whole blood for *B microti* DNA (Focus Diagnostics, San Juan Capistrano, CA). PCR testing for 2 other *Babesia* species were negative. The patient may also have had recent infection with *B burgdorferi*, as suggested by the results of acute-phase serological testing, which showed a Lyme IgM index (also referred to as an immunoglobulin status ratio, or ISR) of 5.64 by enzyme immunoassay (>1.19 is considered positive) and positive IgM Western blot with 2 of 3 bands present (p23 and p39; both Lyme tests done by Laboratory Corporation of America, Dublin, OH). However, false-positive Lyme IgM testing could not be excluded,^7^ given that the IgG Western blot remained negative even during the convalescent phase 8 weeks later (when the IgM Western blot also was negative), although antibiotic treatment could have interfered with evolution of the final antibody results.^8,9^ There was no evidence by IgM serology or PCR of acute coinfection by either *Anaplasma phagocytophilum* or *Ehrlichia chaffeensis*.

## Discussion

**Figure 2.**
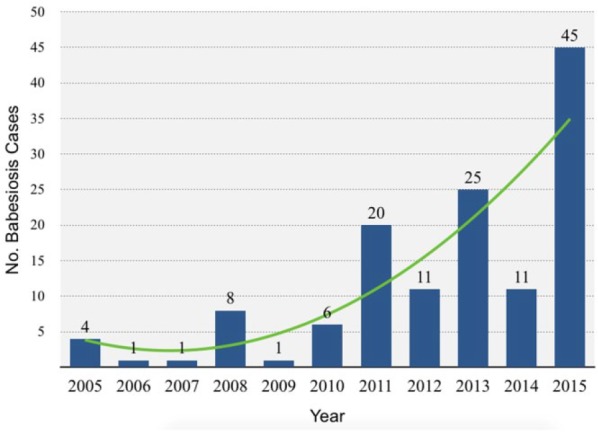
Babesiosis yearly case count in Pennsylvania from 2005 to 2015, including confirmed cases from 2005 to 2010 based on identification of intraerythrocytic *Babesia* organisms on blood smear, and confirmed and probable cases for 2011 to 2015 based on the 2011 CDC case definition.^2^ The true incidence is likely higher because reporting of babesiosis is not mandatory in Pennsylvania.

In the United States, babesiosis is of greatest concern in the Northeast and upper Midwest,^2^ but recent literature suggests expansion into areas previously not considered endemic, such as Maryland, Virginia, and Pennsylvania.^10-12^ The clinical emergence of the *B microti* pathogen in the geographic distribution of its *I scapularis* tick host^13^ might have been predicted, and has been attributed to human encroachment into deer and tick habitats, growth of deer populations, and climatic effects.^11^ Particularly concerning is that this expansion includes several states where reporting of babesiosis is not mandatory,^2^ such as Pennsylvania. For example, a recent analysis of 1363 adult *I scapularis* ticks collected across Pennsylvania found that 3.5% were infected with *B microti* (compared to 47.4% infected with *B burgdorferi* and 3.3% with *A phagocytophilum*), and 2.0% were coinfected by *B microti* and *B burgdorferi*.^14^ Moreover, although the rate of *B burgdorferi* was lower in the western compared to the central or eastern regions of the state, prevalence rates for *B microti* were similar across the state.^14^ The most recent data from the Pennsylvania Department of Health indicate that the number of voluntarily reported cases of human babesiosis increased 10-fold from 2005 to 2015 (Figure 2), although the true incidence is unknown. For the 92 cases reported for 2012 to 2015, 87% were in residents of southeastern Pennsylvania. Unfamiliarity with this pathogen in areas outside those generally recognized as endemic contributes to a low index of suspicion by clinicians.

Our case report underscores how an awareness of babesiosis is becoming important in an ever-increasing geographic area. In particular, 3 cases of babesiosis in patients from Northampton County in eastern Pennsylvania were described in 2013, although that report came from a hospital in the neighboring Lehigh County.^12^ Our patient was from the adjacent Berks County, but presented to a hospital in Lebanon County and ultimately was treated in a hospital in Dauphin County, both of which are in southcentral Pennsylvania. It is noteworthy that these 5 counties (Northampton, Lehigh, Berks, Lebanon, and Dauphin) are aligned east to west, underscoring how clinicians need to be aware of diseases that are expanding into new regions. Indeed, the initial diagnostic considerations for the physicians evaluating this patient were other causes of hemolysis and thrombocytopenia, such as thrombotic thrombocytopenic purpura, but fortunately a manual review of the peripheral blood smear was performed early. The finding of intraerythrocytic parasites enabled institution of targeted antimicrobials and transfer to a referral hospital, where exchange transfusion likely prevented a poor or even fatal outcome.

The severe disease in our patient seemed surprising given the lack of a history of splenectomy or known immunocompromising condition. However, other case reports indicate that *Babesia* infection can cause severe disease in older persons.^4,6,15^ Additionally, it has been suggested that coinfection with *B burgdorferi* (as our patient may have had) can increase the severity or duration of symptoms, particularly of Lyme disease but perhaps of babesiosis as well,^6,16^ although this issue remains unresolved.^17^ Although severe babesiosis certainly can occur in the absence of concurrent Lyme disease, the *I scapularis* vector can transmit *Babesia, Borrelia*, and *Anaplasma*. Thus, guidelines recommend considering co-infections in patients with severe or persistent symptoms.^18^

Whether the transient clinical worsening and apparent initial rise in parasitemia in our patient following institution of antimicrobial therapy directed against *Babesia* was coincidental also is unknown. Current recommendations for treatment of mild disease from babesiosis is atovaquone plus azithromycin, whereas severe disease is treated with clindamycin plus either quinidine or quinine, typically for 7 to 10 days. Exchange transfusion, as was done for our patient, is reserved for patients with high parasitemia (>10%), severe anemia (hemoglobin <10 g/dL), or renal, hepatic, or pulmonary compromise.^1,18,19^

## Conclusions

With recent evidence of geographic expansion of the *B microti* parasite, clinicians should include babesiosis in the differential diagnosis of patients from apparently nonendemic regions who present with anemia, jaundice, and hemolysis. Given the variable disease progression, early recognition (regardless of the severity of symptoms) is critical for expeditiously starting appropriate therapy and, if necessary in severe cases, arranging for exchange transfusion. Additionally, for patients with severe or rapidly progressive *Babesia* infection, empiric treatment for possible *B burgdorferi* or *Anaplasma* co-infection should be considered. Mandatory reporting of babesiosis, especially in states where the disease has been documented, would provide more reliable epidemiologic data and increase awareness of this disease among clinicians.
